# Applications of Antioxidants in Metabolic Disorders and Degenerative Diseases: Mechanistic Approach

**DOI:** 10.1155/2019/4179676

**Published:** 2019-07-29

**Authors:** Mohamed M. Abdel-Daim, Osama S. El-Tawil, Simona G. Bungau, Atanas G. Atanasov

**Affiliations:** ^1^Department of Zoology, Science College, King Saud University, Riyadh 11451, Saudi Arabia; ^2^Pharmacology Department, Faculty of Veterinary Medicine, Suez Canal University, Ismailia 41522, Egypt; ^3^Toxicology and Forensic Medicine, Faculty of Veterinary Medicine, Cairo University, Giza, Egypt; ^4^Department of Pharmacy, Faculty of Medicine and Pharmacy, University of Oradea, Oradea 410028, Romania; ^5^The Institute of Genetics and Animal Breeding, Polish Academy of Sciences, Jastrzębiec, 05-552 Magdalenka, Poland; ^6^Institute of Neurobiology, Bulgarian Academy of Sciences, 23 Acad. G. Bonchev Str., 1113 Sofia, Bulgaria; ^7^Department of Pharmacognosy, University of Vienna, Vienna, Austria

Oxidative stress (resulting from redox homeostasis imbalance between prooxidative and antioxidant systems) is a major player in the pathogenesis of many inflammatory, metabolic, cardiovascular, degenerative, and neoplastic diseases [1]. To counteract this pathological mechanism, exogenous antioxidants act interactively, even synergistically, with the endogenous antioxidant defense system to restore or maintain redox homeostasis [2]. For example, some phytochemicals (e.g., *epigallocatechin gallate*, resveratrol, phytosterol, myricetin, and gingerol) directly influence the numerous pathways of molecular signal transduction (cell proliferation/migration, inflammation cascade, metabolic disorders, and oxidative stress) [3, 4]. Further, many foods in the human diet—vegetables, fruits, juices, and beverages—contain antioxidants [5, 6]. Epidemiological studies have shown that long-lasting consumption of antioxidants through food intake has the potential to protect against multiple diseases, including cancer, diabetes, and neurodegenerative and cardiovascular diseases. In this special issue, several studies described different molecular mechanisms for the alleviation of oxidative stress and the prevention of disorders related to ageing and metabolic and degenerative disorders.

In a cardiovascular research-focused work, I. Peluso and colleagues explored the effects of frequent vegetable consumption on the clinical, antioxidant, and immunological markers in individuals at risk of cardiovascular diseases. J. Li and his team examined the role of the milk fat globule (epidermal growth factor 8) as an antioxidant against neuroinflammation. K. Feng et al. explored the therapeutic effect of curcumin in the anterior cruciate ligament crossing on the osteoarthritis rat model and studied the specific mechanisms by which curcumin inhibits chondrocyte apoptosis, triggered by tertiary butyl hydroperoxide. N. A. Stefanova et al. depicted the suppression of Alzheimer's disease-like pathology progression using mitochondria-targeted antioxidant (SkQ1) in a transcriptome profiling study. Bridging oncology and angiology, M. Alasvand et al. highlighted the effects of some alkaloids on the angiogenesis process that influences cellular invasion and tumor growth.

In the field of ophthalmology, S. Bungău et al. presented an overview of the role, mechanisms, and potential synergistic effects of polyphenols (e.g., anthocyanins, *ginkgo biloba*, resveratrol, and quercetin) and carotenoids (e.g., lutein, zeaxanthin, and mezoxanthin) in the prevention and therapy of age-related ocular pathologies. C.-C. Chang et al. reported that melatonin significantly inhibited H_2_O_2_-induced retinal pigment epithelium (RPE) cell damage and apoptosis, increased the mitochondrial membrane potential, and augmented the autophagy effect. S. Satish et al. concluded that the molecule ZLN005 (a selective PGC-1*α* transcriptional regulator) increases PGC-1*α* expression in the human RPE and protects cells against death by three major biological prooxidants. In the same study, they demonstrated that PGC-1*α* is the critical mediator of ZLN005 antioxidant effects.

In a diabetes research-focused work, A. E. Zayed et al. explored the protective effects of *Ginkgo biloba* and magnetised water against nephrotoxicity associated with type 2 diabetes mellitus in rats. In the same vein, R. Jimenez et al. investigated the value of Nrf2 signalling in peroxisome proliferator-activated receptor (PPAR)*β*/*δ*-mediated vascular protection against hyperglycemia-induced oxidative stress. They concluded that PPAR*β*/*δ* agonists can downregulate the Nrf2 pathway, suggesting a possible therapeutic role for PPAR*β*/*δ* in diabetic vascular complications. A. M. Papinska and K. E. Rodgers demonstrated that the administration of angiotensin (1–7) [A(1–7)] to a mouse model of severe type 2 diabetes (db/db) prevented the formation of nitrotyrosine residues and reduced the expression of the two enzymes (eNOS and NOX-4) involved in nitrotyrosine formation. M. B. Alam et al. characterized the multiple actions of gossypol from cottonseeds showing that it mimics insulin, inhibits the activity of *α*-glucosidase, accelerates glucose uptake in C2C12 myotubes, amplifies the expression of glucose transporter-4 in the muscle tissue, and suppresses gluconeogenesis in the liver of streptozotocin-induced diabetic mouse model. R. Wang et al. presented their research about the endogenous cystathionine *γ*-lyase/hydrogen sulfide system which regulates the effects of insulin and glucocorticoids on muscle protein synthesis.

Regarding hepatoprotection, O. M. Ahmed et al. demonstrated that the hydroethanolic extracts of orange, naringin, and naringenin have hepatopreventive effects in rats by stimulating the antioxidant defense system and suppressing inflammation and apoptosis. Aiming at a possible treatment strategy for fatty nonalcoholic liver disease, Q. Chu et al. highlighted the effects of cervical anthocyanins on the hepatic accumulation of oleic acid by activating autophagy. Meanwhile, L. M. França and colleagues focused on understanding the molecular mechanisms by which the hydroethanolic extract of *Syzygium cumini* leaves alleviates monosodium L-glutamate-induced hypertriglyceridemia in obese rats. The results of another study by H. Farghali et al. showed that SIRT1 plays a role in the hepatoprotective effects of polyphenols; SIRT1 allosteric activators mimic the hepatoprotective effects of polyphenols, and the pharmacological modulation of SIRT1 by STACs may be a future major step in the treatment of xenobiotic-induced hepatotoxicity.

In the same field, T. Albrahim and M. A. Binobead showed how *Moringa* leaf extract could ameliorate the biochemical changes, oxidative stress, hepatic injury, and PCNA and P53 alterations, induced by vetsin (monosodium glutamate) administration. Moreover, W. Tang et al. demonstrated the potential of Hugan Qingzhi tablet (a lipid-lowering and anti-inflammatory formula) in preventing and treating fatty nonalcoholic liver diseases in rats, along with its modulatory effect on the intestinal microbiota. Similarly, M. A. Dkhil et al. studied the effects of *Indigofera oblongifolia* leaf extract (IE) on the hepatic oxidative status, as well as the expression of apoptotic and inflammatory genes in blood-stage murine malaria. They concluded that IE protects the liver tissue from damages caused by *P. chabaudi*, via anti-inflammatory and antioxidant mechanisms.

In the field of neuromuscular disease, P. Xu et al. demonstrated that after transplantation, the overexpression of BRCA1 in the neural stem cells enhances functional recovery and cell survival into the experimental ischemic stroke, reducing oxidative stress and cell apoptosis. Further, O. V. Yakovleva et al. tested the beneficial action of the H_2_S donor (NaHS) on the redox state, physical development, locomotion and exploratory activity, muscle strength, reflex ontogeny, and motor coordination of pups with maternal hyperhomocysteinemia.

Regarding hormonal therapies, A. B. Abdel-Naim et al. reported the beneficial effects of 2-methoxyestradiol in attenuating testosterone-induced benign prostatic hyperplasia in experimental rats, partly via inhibition of the HIF-1*α*/TGF-*β*/Smad2 axis. H. M. A. Abdelrazek et al. proved that hormone replacement therapy by food/therapeutic intake of soy isoflavones improves metabolic and immunological changes (lipid profile, loss and bone mineralization, and appetite) that occur after ovariectomy in Wistar rats. In addition, J. J. Lim et al. suggested tocotrienol-rich fraction treatment for the modulation of satellite cell renewal by regulating gene expressions, i.e., p53 signalling (RRM2B and SESN1), cell cycle, Wnt signalling pathways (a group of signal transduction), and myogenic regulatory factor expression. J. Wattanathorn and his team studied the effects of the encapsulated mulberry fruit extract on the changes that occur in metabolic syndrome in a menopausal animal model.

Finally, A. W. K. Yeung et al. conducted an extensive study on the antioxidant literature, identifying and analyzing the relevant publications (299,602 manuscripts using VOSviewer). Most of the identified articles were published after 1991. The analysis performed by the authors revealed a shift in scientific interest from vitamins/minerals with antioxidant action to antioxidant phytochemicals.

Based on the works mentioned above, the main objective of many studies remains to understand how oxidants act on molecular targets and the pathogenesis of related diseases. The results of these researches are aimed at characterizing novel preventive interventions and suggesting optimal therapeutic schemes.
